# Analgesic Characteristics of NanoBEO Released by an Airless Dispenser for the Control of Agitation in Severe Dementia

**DOI:** 10.3390/molecules27154987

**Published:** 2022-08-05

**Authors:** Damiana Scuteri, Laura Rombolà, Takafumi Hayashi, Chizuko Watanabe, Shinobu Sakurada, Kengo Hamamura, Tsukasa Sakurada, Paolo Tonin, Giacinto Bagetta, Luigi A. Morrone, Maria Tiziana Corasaniti

**Affiliations:** 1 Pharmacotechnology Documentation and Transfer Unit, Preclinical and Translational Pharmacology, Department of Pharmacy, Health and Nutritional Sciences, University of Calabria, 87036 Rende, Italy; 2 Regional Center for Serious Brain Injuries, S. Anna Institute, 88900 Crotone, Italy; 3 Preclinical and Translational Pharmacology, Department of Pharmacy, Health Science and Nutrition, University of Calabria, 87036 Cosenza, Italy; 4 Laboratory of Pharmaceutical Sciences, Faculty of Pharmaceutical Sciences, Tohoku Medical and Pharmaceutical University, Sendai 981-8558, Japan; 5 Department of Physiology and Anatomy, Faculty of Pharmaceutical Sciences, Tohoku Medical and Pharmaceutical University, Sendai 981-8558, Japan; 6 Center for Clinical Pharmacology and Pharmaceutics, Nihon Pharmaceutical University, Saitama 362-0806, Japan; 7 Center for Supporting Pharmaceutical Education, Faculty of Pharmaceutical Sciences, Daiichi University of Pharmacy, Fukuoka 815-8511, Japan; 8 Department of Health Sciences, University “Magna Graecia” of Catanzaro, 88100 Catanzaro, Italy

**Keywords:** essential oil of bergamot, nanotechnology delivery system, NanoBEO, dementia, pain, NPS, agitation

## Abstract

Chronic pain is one of the most common causes of the need for clinical evaluation, acquiring more importance in the elderly with cognitive impairment. Reduced self-reporting capabilities cause unrelieved pain contributing to the development of agitation. Safe and effective pain treatment can afford the management of agitation without the serious increase in death risk associated with neuroleptics. To this aim, the essential oil of bergamot (BEO), proven by rigorous evidence to have strong preclinical anti-nociceptive and anti-allodynic properties, has been engineered (NanoBEO, patent EP 4003294) to allow randomized, double-blind, placebo-controlled trials (BRAINAID, NCT04321889). The present study: (1) assesses the analgesic effects of a single therapeutic dose of NanoBEO, as supplied by an airless dispenser for clinical translation, in models of inflammatory, neuropathic, and sensitization types of pain relevant to clinic; (2) provides a dose–response analysis of the efficacy of NanoBEO on scratching behavior, a typical behavioral disturbance occurring in dementia. A single therapeutic dose of NanoBEO confirms efficacy following thirty minutes pre-treatment with capsaicin and on the central sensitization phase induced by formalin. Moreover, it has an ID50 of 0.6312 mg and it is efficacious on static and dynamic mechanical allodynia. Altogether, the gathered results strengthen the potential of NanoBEO for clinical management of pain and agitation.

## 1. Introduction

Patients often come to clinical observation because of chronic pain [1], among which low back pain [2] is one of the most disabling conditions, with a global lifetime prevalence of about 39% [3]. Chronic pain acquires more importance in the elderly [4,5] for several reasons. This fragile population most often experiences chronic pain due to age-related comorbidities, such as diabetes [6] and shingles due to herpes zoster infection [7], but also injury [8], stroke [9], and rheumatic conditions, usually including neuropathic features [10,11,12]. Aging influences pain and sensitization processes as well as the effectiveness of commonly used pharmacological agents [13]. Aged people are often unjustifiably excluded from clinical trials [14], particularly in migraine research [15,16,17], thus preventing the accurate knowledge of the efficacy and safety of painkillers in these patients accounting for pathophysiological variability [18] and polypharmacy [19]. People aged over 65 are often affected by dementia and the pain conditions that they experience are usually underdiagnosed due to their reduced and insufficient self-report skills [20]. This unrelieved pain contributes to the development of the most challenging neuropsychiatric symptoms (NPS), i.e., agitation [21]. Patients suffering from cognitive impairment receive a significantly smaller amount of analgesic drugs and with reduced dosage [22], even in the community setting causing an increase in potentially harmful psychotropic treatments [23]. Agitation and pain control with essential oils endowed with a sound rationale for clinical translation [24] have never been investigated so far. The essential oil of bergamot (BEO, s) has proven strong preclinical evidence of anti-nociceptive and anti-allodynic effects and has undergone a pharmacotechnological process to allow its delivery in known amounts through α-tocopheryl stearate solid lipid nanoparticles (α-TFS-SLN), with physicochemical stability and without smell [25] to permit the masking of clinical trials [26]. In fact, phytonanotechnology presents a new scenario, allowing several applications in medical fields, e.g., the development of a new class of nanoantibiotics to manage multi-drug resistance [27]. In particular, several plant secondary biomolecules absent in bacteria reduce metals, causing the production of differently sized nanoparticles with applications ranging from medicine, biology, material science, physics, and chemistry to agriculture; moreover, metal oxides synthesized through different parts/extracts of the plants confer biofunctionalization ranging from antibacterial, antioxidant, anticancer, antifungal to cytotoxic activity [27]. Nanoantibiotics can cause DNA damage, oxidative damage, and cell wall and membrane damage through oxidative stress. The properties of the novel metal nanoparticles are greatly influenced by their size, shape, composition, crystallinity, and structure [27]. Within the exploding field of research about nanoparticles, the present study intends to: (1) verify that a single therapeutic dose of the nanotechnology-based delivery system NanoBEO (patent EP 4003294), as supplied by the airless dispenser for clinical translation, confirms the anti-nociceptive and anti-allodynic properties of BEO in models of inflammatory, neuropathic, and sensitization type of pain relevant to chronic pain in the elderly; (2) provide a dose–response analysis of NanoBEO efficacy on scratching behavior, a typical NPS occurring in dementia.

## 2. Results

### 2.1. Confirmation of Composition of NanoBEO Cream in the Dispenser

**Figure 1:**
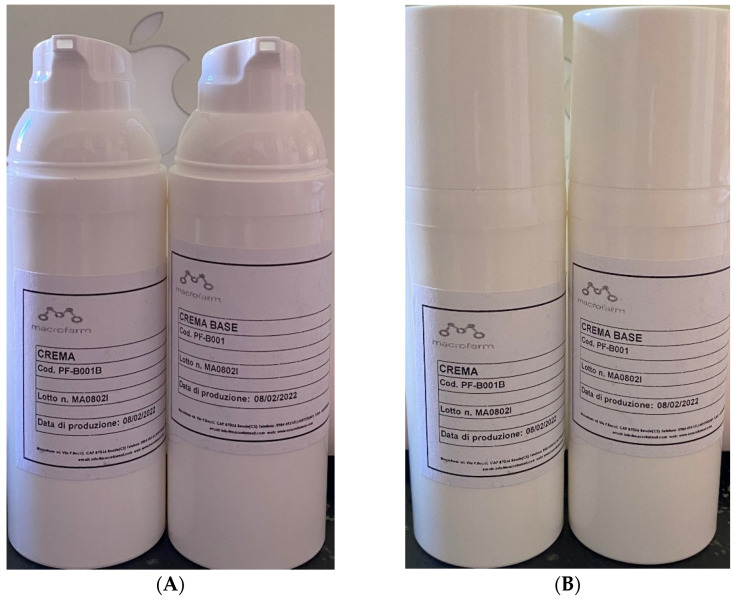
(**A**) Airless dispensers containing NanoBEO and control cream, (**B**) covered with cap.

After dilution of a volume of 1 mL of the α-TFS-SLN formulation with methanol and analysis by spectrophotometric detection at wavelengths of 281 nm for linalool, 208 nm for linalyl acetate, and 247 nm for limonene, the following composition of 44.227 g of cream is confirmed: 37.604 g of purified water suspension of α-TFS-SLN containing the whole BEO (α-Pinene 0.7–2.0; Sabinene 0.5–2.0; β-Pinene 5.0–10.0; Limonene 30.0–50.0; γ-Terpinene 6.0–18.5; Linalool 6.0–15.0; Linalyl acetate 23.0–35.0; Geranial < 0.5; Geranyl acetate 0.1–0.7; Cariophyllene 0.2–0.5), defurocumarinized to avoid phototoxicity; 4.42 g of sweet almond oil; 0.885 g of polyacrylamide; 0.442 g of isoparaffin C13–14; 0.111 g of 7-laurate; 0.774 g of purified water Ph.Eur.; 0.028 g of methyl paraben; 0.009 g of propylparaben. The pH regulation due to levels of CO_2_ is fundamental for the correct function of the human proteome [28]. The pH of NanoBEO cream is 5.72, thus very similar to that of the skin, i.e., 5.5 [29]. This is noteworthy because SLN could undergo aggregation in the presence of electrolytes at neutral or lower pH and the topical environment for administration is simulated with pH 5.5 [30]. Therefore, once in the airless dispenser (Figure 1A,B), one dose from the dispenser contains 4.4 g of cream for the clinical trial. An equal dose of empty cream was used as a control.

### 2.2. Effect of Single Therapeutic Dose of NanoBEO on Capsaicin-Induced Nociceptive Behavior

**Figure 2:**
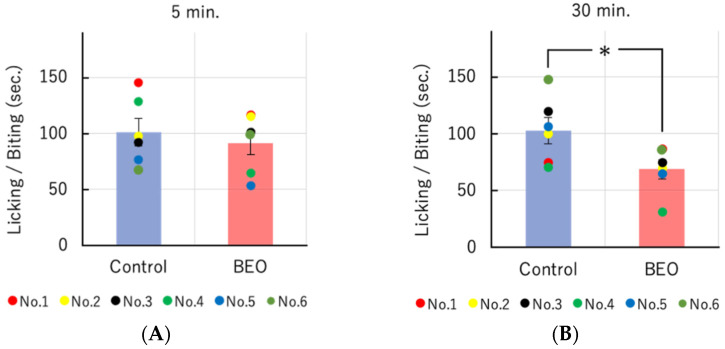
Effect of transdermal administration of a single therapeutic dose of NanoBEO on capsaicin-induced licking/biting behavior. (**A**) The duration of licking/biting induced by intraplantar injection of capsaicin (1.6 μg/20 μL) is determined using the 5-min period starting immediately after injection of capsaicin. (**B**) NanoBEO reduces significantly capsaicin-induced licking/biting behavior after 30 min. Empty cream is used as a control and it fails to affect capsaicin-induced nociceptive response. The data presented are expressed as mean ±S.E.M. (*n* = 6). The value of * *p* < 0.05 is considered statistically significant.

The efficacy of one supply of NanoBEO as distributed by the dispenser on the number of seconds (sec) of licking/biting behavior after 5 min (min) and 30 min after capsaicin administration confirms the anti-nociceptive effect of NanoBEO. Empty cream, used as a control, fails to affect capsaicin-induced nociceptive response (Figure 2A,B).

### 2.3. Effect of Single Therapeutic Dose of NanoBEO on Formalin-Induced Biphasic Behavior

**Figure 3:**
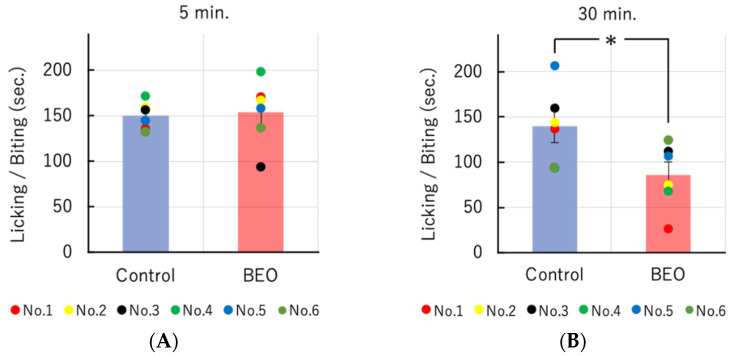
Effect of transdermal administration of a single therapeutic dose of NanoBEO on 0.5% formalin-induced biphasic licking/biting behavior. (**A**) The duration of licking/biting induced by intraplantar injection of 0.5% formalin is determined using the 5-min period beginning immediately after the administration of formalin. (**B**) NanoBEO reduces significantly formalin-induced central sensitization after 30 min. Empty cream is used as a control and it fails to affect formalin-induced nociceptive response. The data presented are expressed as mean ±S.E.M. (*n* = 6). The value of * *p* < 0.05 is considered statistically significant.

The efficacy of one supply of NanoBEO as distributed by the dispenser on the number of seconds (sec) of licking/biting biphasic behavior induced by formalin confirms the significant effectiveness of BEO in the central sensitization phase occurring 30 min after the injection of formalin. Empty cream, used as a control, does not prove efficacy in the formalin test (Figure 3A,B).

### 2.4. Effect of Single Therapeutic Dose of NanoBEO on PSNL-Induced Static and Dynamic Allodynia

**Figure 4:**
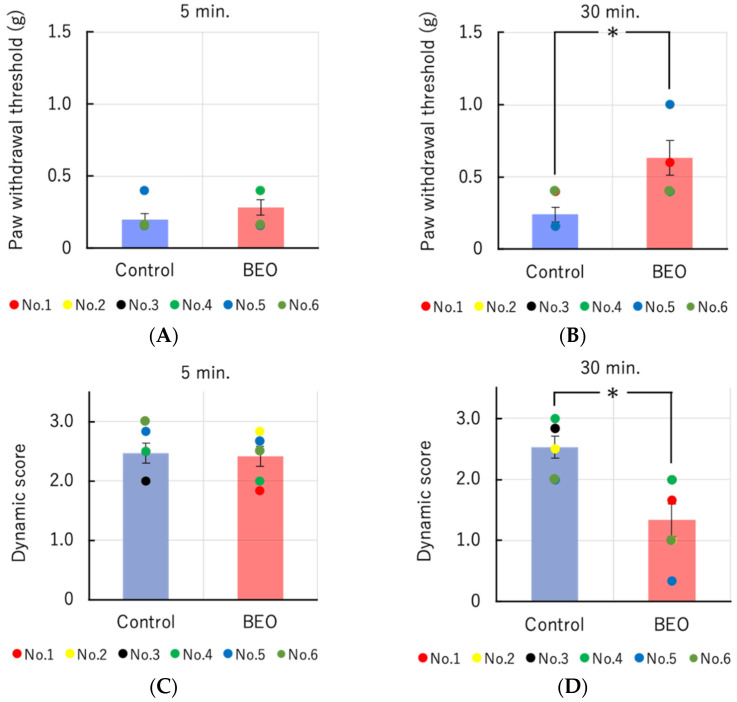
Effect of transdermal administration of a single therapeutic dose of NanoBEO on static (**A**,**B**) and dynamic (**C**,**D**) mechanical allodynia induced by partial sciatic nerve ligation (PSNL). NanoBEO reduces significantly static and dynamic mechanical allodynia occurring 30 min after the beginning of the test. Empty cream, used as a control, does not prove efficacy on both types of allodynia. The data presented are expressed as mean ±S.E.M. (*n* = 6). The value of * *p* < 0.05 is considered statistically significant.

On the 7th post-operative day after induction of neuropathic pain through PSNL, static mechanic allodynia is assessed through Von Frey’s hairs, while dynamic allodynia is evaluated by light stroking of the plantar surface of the hind paw from the toe of the hind paw with an art paint-brush, ranking responses as follows: 0, no response; 2, lifting of the stimulated hind paw; 3, flinching or licking of the stimulated hind paw. Baseline response scores are determined before PSNL and on post-operative day 7. The efficacy of a single therapeutic dose of NanoBEO as supplied by the dispenser to increase the paw withdrawal threshold is statistically significant on static and dynamic mechanical allodynia occurring 30 min after the beginning of the test. Empty cream, used as a control, does not prove efficacy on both types of allodynia (Figure 4A–D).

### 2.5. Dose–Response of NanoBEO on 4-Methyl Histamine-Induced Scratching Behavior

**Figure 5:**
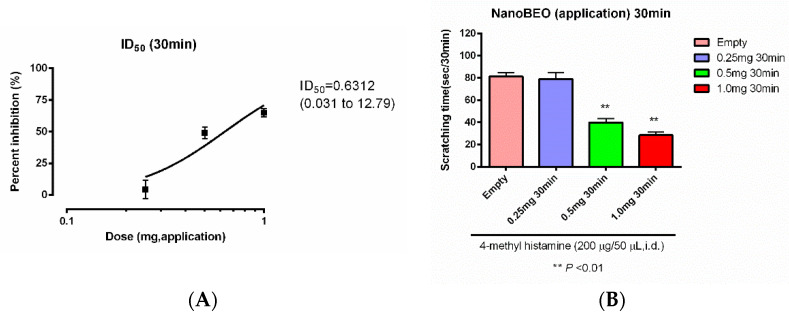

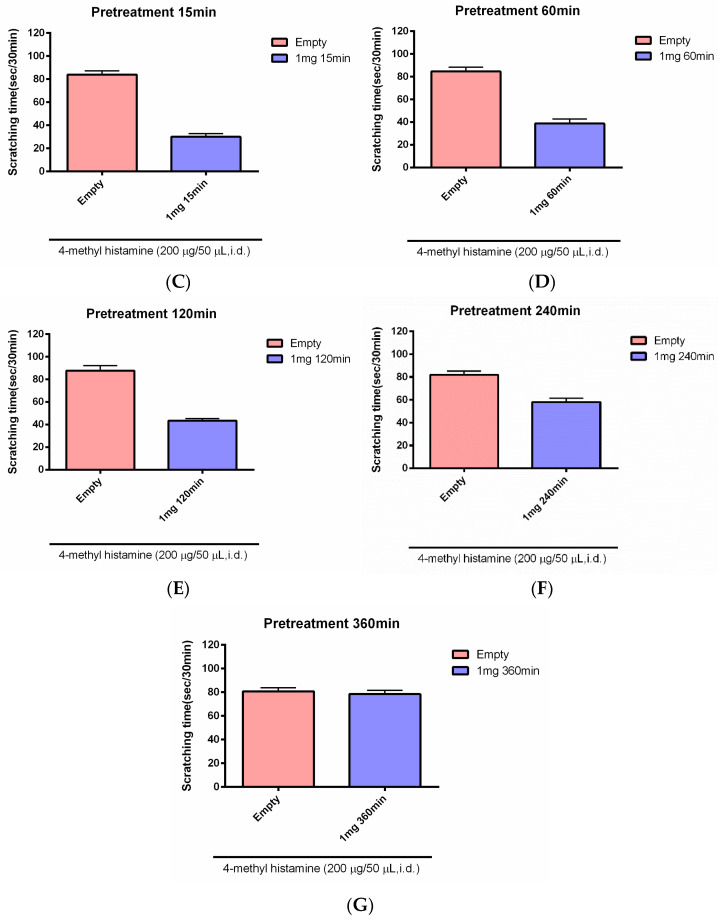
Dose–response (**A**,**B**) and time course (**C**–**G**) of the efficacy of NanoBEO on 4-methyl histamine-induced scratching behavior. The effectiveness of NanoBEO increases with the dose, reaching statistical significance at 0.5 and 1 mg. The effect is not statistically significant when pre-treatment is performed at the following time points: 15 min, 60 min, 120 min, 240 min, and 360 min. Each value represents the mean ± S.E.M. of *n* = 6 mice. The value of *p* < 0.05 is considered statistically significant. ** *p* < 0.01.

Mice are pre-treated thirty min before the intradermal administration of 4-methyl histamine with NanoBEO 0.25, 0.50, and 1 mg (Figure 5A), proving enhanced efficacy with the increase of the dose, statistically significant at 0.50 and 1 mg (Figure 5B). The inhibition dose (ID)50 is 0.6312 mg. The effect is not statistically significant when pre-treatment is performed at the following time points: 15 min, 60 min, 120 min, 240 min, and 360 min (Figure 5C–G).

## 3. Discussion

Of the various essential oils investigated for analgesic effectiveness, BEO provides the strongest preclinical evidence to justify clinical investigation according to the guidelines for Animal Research: Reporting In Vivo Experiments (ARRIVE) [31], the Systematic Review Center for Laboratory Animal Experimentation (SYRCLE’s) risk of bias (RoB) tool [32], and the Collaborative Approach to Meta-Analysis and Review of Animal Data from Experimental Studies (CAMARADES) checklist for study quality [33]. The present study provides an exact single therapeutic dose for clinical trial supplied from an airless dispenser containing 4.4 g of cream. Moreover, it allows us to know the ID50 of NanoBEO which is 0.6312 mg. In fact, the effect of the nanotechnological formulation on 4-methyl histamine-induced scratching behavior increases with the dose, reaching statistical significance at 0.50 and 1 mg. Interestingly, the effect needs at least 30 min to occur, after which it starts to decrease, in agreement with preclinical findings on the different fractions of BEO [34,35]. The single therapeutic dose of NanoBEO confirms its efficacy in the acute phases of nociceptive models in the capsaicin test and the first phase of the formalin test [36], but also in the sensitization of the formalin test. This test is of the utmost importance as a model for chronic pain in the elderly, since its pattern is influenced by aging [37,38] and it is characterized by mechanisms of central sensitization occurring at the level of the dorsal horn [39] and implicated in the late phase and long-term mechanical allodynia induced by formalin [40,41]. Furthermore, the nocifensive reaction associated with formalin injection has been found to include neuropathic features [42]: it induces concentration-dependent hypersensitivity with an increase of voltage-gated L-type calcium channel α2δ − 1 subunits in dorsal root ganglia, i.e., a marker of neuropathic pain; mechanical allodynia produced by formalin responds to gabapentin, targeting α2δ − 1 subunit, paralleling spinal nerve injury-caused allodynia. NanoBEO proves efficacy not only on static allodynia induced by PSNL, but also on dynamic allodynia, that is evoked by tangential movement across the skin [43]. This characteristic, together with the anxiolytic-like activity on a serotonergic basis, is fundamental for the control of agitation [44,45], and devoid of the sedation that could aggravate cognitive impairment [44] making NanoBEO a very suitable candidate tool for the safe and effective management of NPS in dementia. In order to allow the accurate assessment of pain in patients suffering from severe dementia in the clinical trial to investigate the clinical efficacy of NanoBEO, the Italian version of the Mobilization-Observation-Behavior-Intensity–Dementia, the I-MOBID2, has been recently validated in the Italian setting [46] and any herbal drug interactions deserve future evaluation [47].

## 4. Materials and Methods

### 4.1. Reagents

Solvents were bought from Sigma-Aldrich (Sigma Chemical Co., St. Louis, MO, USA): tetrahydrofuran (THF), chloroform (CHCl3), n-hexane, ethyl acetate, dimethyl sulfoxide (DMSO), isooctane, 1-butanol, α-linolenic acid (PM = 278.43 g/mol), α-tocopherol (PM = 430.72 g/mol), biliary salt of taurodeoxycholic acid, Tween 20, and dicyclohexylcarbodiimide (DCC).

### 4.2. Essential Oil and NanoBEO

BEO, furocoumarin-free to prevent phototoxicity [48], was kindly provided by “Capua Company 1880 s.r.l.”, Campo Calabro, Reggio Calabria (Italy). The certificate of analysis confirms its composition in percentage (%): α-Pinene 0.7–2.0; Sabinene 0.5–2.0; β-Pinene 5.0–10.0; Limonene 30.0–50.0; γ-Terpinene 6.0–18.5; Linalool 6.0–15.0; Linalyl acetate 23.0–35.0; Geranial < 0.5; Geranyl acetate 0.1–0.7; Cariophyllene 0.2–0.5. The essential oil was encapsulated in α-TFS-SLN synthesized as previously described, using a microemulsion technique at a moderate temperature [49,50]. In particular, α-TFS (142 mg, 0.201 mmol) was mixed with BEO furocoumarin-free by heating at a temperature in the range of 60–65 °C to avoid BEO degradation. Sodium taurodeoxycholate and Tween 20 were used as emulsifiers and butanol as a co-emulsifier and microemulsion underwent immediate dispersion in cold water (1:20; 2 °C) under high-speed homogenization (Model SL2, Silverson, Chesham Bucks, UK) at 8000 rpm for 30 min (240.000 g in 30 min). Dispersions were washed twice using an Amicon TCF2A ultrafiltration system (Amicon Grace, Beverley, MA, USA; membrane Amicon Diaflo YM 100). The nanotechnology delivery system consists of an airless dispenser delivering a fixed amount of a cream incorporating the α-TFS-SLN containing BEO devoid of furocoumarins. BEO was encapsulated in SLN with anti-oxidant components in order to: afford stability and titration of the active components; allow reproducibility of data; obtain an odorless cream indistinguishable from the placebo, and perform double-blind clinical trials [25]. One supply from the dispenser contained 4.4 g of cream. An equal dose of empty cream was used as a control. The transdermal administration of the cream occurred through a cotton swab followed by massage up to complete absorption in the inter-shoulder region, after measuring that the mouse could not reach the site with the hind paw in any projection to prevent its licking. The complete airless dispenser containing NanoBEO and empty control cream was produced by the spin-off of the University of Calabria “Macropharm s.r.l.”, Via P. Bucci, Rende (Italy).

### 4.3. Animals

The experiments were conducted using male ddY (SD) mice (Shizuoka Laboratory Center, Japan; Japan SLC, Hamamatsu, Japan; Kyudo Industries, Kumamoto, Japan) weighing 22–26 g. The mice were individually housed and subjected to 12 h light/dark cycle, room temperature 23 °C, 50–60% relative humidity with food and water *ad libitum*. To prevent behavioral changes due to circadian rhythm, all the experiments were carried out between 10:00 and 17:00 h in a quiet room, randomizing the order of tests. The study follows the approval of the Ethics Committee for Animal Experiments of the Daiichi University of Pharmacy and Tohoku Pharmaceutical University (Examination number of Daiichi University of Pharmacy: H29-005, approval number: 17003), and the National Institutes of Health Guide for the Care and Use of Laboratory Animals [51]. In agreement with the 3R approach to refine, reduce, and, at least in part, replace, a statistical power analysis was calculated on similar studies in the literature finding *n* = 5 for obtaining a 30% reduction in nociceptive reaction. To prevent variability due to the different experimental conditions, before every behavioral test, each mouse was acclimatized to an acrylic observation chamber (22.0 × 15.0 × 12.5 cm) for approximately 1 h. All the tests were performed by a blind observer.

### 4.4. Capsaicin and Formalin Test

Mice were subjected to the capsaicin test [52,53] and to the formalin test [54], to observe the effect of one dose of NanoBEO on the acute and biphasic responses with sensitization, respectively. The right hind paw was i.pl. administered 20 μL of a solution of capsaicin (1.6 μg/20 μL) and 20 μL of formalin (0.5% in saline) through a 50 μL Hamilton microsyringe with a 26-gauge needle, with strictly necessary animal restraint, in each test respectively. Thirty min before capsaicin/formalin injection, one supply of NanoBEO was transdermally administered through a cotton swab. Mice were placed in the test box for a period of observation of the number of seconds of licking/biting with a hand-held stopwatch 5 and 30 min after the administration of capsaicin/formalin.

### 4.5. PSNL

Mice were anesthetized using isoflurane (2.0%, FUJIFILM Wako Pure Chemical Corporation, Osaka, Japan). The sciatic nerve of the right hindlimb was exposed at high thigh level through a small incision and the distal one-third to one-half of the dorsal portion of the sciatic nerve was tied with non-absorbable silk thread. A supply of NanoBEO and control from an airless dispenser was transdermally applied on the 7th post-operative day, thirty min before the evaluation of mechanical allodynia. The presence of mechanical allodynia was assessed by the Von Frey’s test after 1 h of acclimation in a plexiglass observation chamber (9.0 × 9.0 × 14.0 cm, length × width × height, Ugo Basile, Gemonio, Italy) with a wire mesh floor, using calibrated Von Frey’s filaments (pressure stimulus 0.40 g, Natsume Seisakusho Co., Ltd., Tokyo, Japan). In fact, these hairs are characterized by logarithmically incremental stiffness (0.41, 0.70, 1.20, 2.00, 3.63, 5.50, 8.50, and 15.10 g). The paw withdrawal threshold was measured using the up-down method [55].

### 4.6. Scratching Behavior

Scratching is one of the neuropsychiatric symptoms affecting patients suffering from dementia. NanoBEO and control from the airless dispenser were transdermally applied thirty min prior to the intradermal administration of 4 methyl-histamine (200 µg/50 µL) and the scratching behavior was filmed for 30 min and measured offline by an independent observer to provide a dose–response curve (0.25–0.5–1 mg of NanoBEO) with a calculation of inhibition dose (ID)50. 4-methyl-histamine is a pharmacological tool used to induce itching behavior in mice. Pre-treatment was performed at the following time points: 15 min, 60 min, 120 min, 240 min, and 360 min, to assess the duration of the effect of a single therapeutic dose.

### 4.7. Statistical Analysis

The nociceptive response is expressed as the mean± S.E.M. of the seconds of licking/biting in the capsaicin and formalin tests, of the scratching time in the itch test, and of the paw withdrawal threshold for the Von Frey’s test after PSNL (for the error bars calculation *n* = 6 is used). Standard error of the mean has been used for inferential statistics, hence representing a measure of how variable the mean will be if the whole study was repeated six times [56]. The results were subjected to statistical analysis using Student’s *t*-test (GraphPad Prism; GraphPad Software, Inc., San Diego, CA, USA) and considering values of *p* < 0.05 statistically significant.

## Data Availability

The data presented in this study are available within the article.

## Sample Availability

Samples of the compounds are available from the authors.
